# Ectoine lozenges in the treatment of acute viral pharyngitis: a prospective, active-controlled clinical study

**DOI:** 10.1007/s00405-019-05324-9

**Published:** 2019-02-09

**Authors:** Van-Anh Dao, Sabrina Overhagen, Andreas Bilstein, Carina Kolot, Uwe Sonnemann, Ralph Mösges

**Affiliations:** 1 CRI-Clinical Research International Ltd., Cologne, Germany; 2 bitop AG, Dortmund, Germany; 3 Private Health Centre, Institute for ENT Elmshorn, Elmshorn, Germany

**Keywords:** Acute pharyngitis, Ectoine, Ectoine lozenges, Sore throat

## Abstract

**Purpose:**

Acute pharyngitis is an uncomfortable disorder mostly caused by viruses and for which antibiotics are unwarranted. This study compared lozenges containing ectoine, a natural extremolyte, with hyaluronic acid lozenges and hypertonic saline gargle for symptomatic treatment of acute viral pharyngitis.

**Methods:**

This prospective, controlled clinical study, recruited 90 patients with moderate-to-severe pharyngitis symptoms who chose to use either ectoine (*n* = 35), hyaluronic acid (*n* = 35), or saline gargle (*n* = 20). Patients applied their 7-day treatment from the inclusion visit (V1) until the end-of-study visit (V2). Patients’ pharyngitis symptoms, general health, general treatment effectiveness and tolerability, and patient compliance were assessed by investigators and patients.

**Results:**

The sum score for three primary symptoms (pain on swallowing, urge to cough, and hoarseness) decreased by 79.5% (ectoine), 72.2% (hyaluronic acid), and 44.8% (saline gargle). Both lozenges were significantly superior to saline gargle (*P* < 0.05). Regarding general health improvement, ectoine was significantly superior to saline gargle (72.5% vs. 45.2%, *P* < 0.05), but hyaluronic acid (63.3%) was not. At V2, 65.7% of patients receiving ectoine reported “very good” general health vs. 48.6% of those receiving hyaluronic acid and 20.0% using saline gargle. Ectoine was significantly superior (*P* < 0.05) to both hyaluronic acid and saline gargle in terms of tolerability and patient compliance. No patients taking ectoine reported unpleasant sensations while applying their treatment, whereas almost half of patients using hyaluronic acid lozenges and saline gargle did.

**Conclusion:**

Treatment with ectoine lozenges significantly relieves moderate-to-severe symptoms of acute viral pharyngitis and is more effective and tolerable than treatments with hyaluronic acid lozenges and hypertonic saline gargle.

**Electronic supplementary material:**

The online version of this article (10.1007/s00405-019-05324-9) contains supplementary material, which is available to authorized users.

## Introduction

Acute pharyngitis is a highly prevalent community-acquired infection characterised by a sore throat. This disorder is mostly caused by viruses (i.e., uncomplicated, self-limited acute pharyngitis), is generally treated symptomatically [1], and, therefore, does not require antibiotics. The only common type of acute pharyngitis that calls for antibiotics involves group A *Streptococcus* [2], representing about 10% of all cases in adults [3]. However, despite decades of efforts to reduce the rate of antibiotic prescription for acute pharyngitis, it is currently about 60% [4, 5], far exceeding what is clinically justified. In acute pharyngitis, patients’ expectation of antibiotics is an important force driving doctors to prescribe them, but patients who ask for antibiotics mostly just want pain relief [6]. On the whole, antibiotics improve symptoms and duration of disease modestly [7], and the risk of developing complications (e.g., acute rheumatic fever, quinsy) is not demonstrably lower in patients treated with antibiotics than in those without, at least in Western countries [8]. Unnecessary prescription of antibiotics is not benign—it fuels antibiotic resistance, a global health, and food security threat. The effectiveness of antibiotics should be preserved for generations to come; to this end, a treatment that can provide pharyngitis patients with effective symptom relief would constitute a strong argument against antibiotic overuse.

In the last decade, the bacteria-derived extremolyte ectoine has attracted considerable medical interest. Evidence for the inflammatory-reducing effect of ectoine from preclinical studies [9–15] has heralded its application in the treatment of dermatitis, allergic rhinitis, sinusitis, mucositis, and lung inflammation [16–22]. Ectoine acts via a mechanism shared among extremolytes known as “preferential exclusion” [23], which means that it is preferentially excluded from the water–protein interface. By enhancing the hydrogen bond properties, ectoine forms a virtual water capsule around proteins [24, 25]. This mechanism of action grants ectoine the ability to stabilise protein and biomembrane structures [26, 27].

In our previous work [28], we demonstrated the superiority of a mouth and throat spray containing ectoine over saline lozenges in the treatment of mild-to-moderate acute pharyngitis and/or laryngitis. In this study, we investigated lozenges containing ectoine in patients with moderate-to-severe symptoms of acute pharyngitis. The lozenges’ effectiveness, tolerability, and safety were compared to those of two active controls, hyaluronic acid lozenges and hypertonic saline gargle.

## Materials and methods

### Study design

This prospective, active-controlled clinical study was conducted in three ear, nose, and throat surgeries in Germany from January to May 2016. It was approved by the *Freiburger Ethik-Kommission International* (“freiburg ethics commission international”, Freiburg, Germany; reference number 016/1014) and registered at ClinicalTrials.gov under the identifier NCT02669446. Before being included, all patients signed an informed consent form allowing the use of their data in this study.

Since the investigational product is a medical device that had already been granted marketing authorisation, this study was designed in accordance with the German Medical Devices Act (Medizinproduktegesetz, MPG) Section 23b. This study design aimed to collect data on the use of the investigational product in routine medical practice without randomisation or placebo control. The investigational product was compared with two popular treatment modalities for pharyngitis: over-the-counter hyaluronic acid-containing lozenges and the home remedy hypertonic saline gargle. Patients were allowed to choose one of the three treatments before inclusion and after having been informed thoroughly by the investigators about the study and all aspects of the three treatment options.

The treatment lasted for 7 days. The study comprised an inclusion visit (V1) on day 1 and an end-of-study visit (V2) on day 7.

### Patients

A total of 90 female and male patients were to be enrolled in three treatment groups: ectoine lozenges (35 patients), hyaluronic acid lozenges (35 patients), and saline gargle (20 patients). Inclusion criteria were ≥ 18 years of age, diagnosis of acute viral pharyngitis by an investigator, and moderate-to-severe symptoms of pain on swallowing, urge to cough, and hoarseness (see “Clinical assessment” for the defined symptom scores). Exclusion criteria were < 18 years of age, pregnancy, sore throat persisting longer than 5 days, intolerance to ectoine or hyaluronic acid, previous surgery of the pharynx or oral cavity, or suspected bacterial tonsillitis or pharyngitis.

### Study treatments

Ectoine lozenges (Ectoin^®^ Soft Lozenges Wildberry, bitop AG, Dortmund, Germany) are registered medical devices class I indicated for the treatment and prevention of common cold symptoms, dry mouth, and throat and hoarseness due to voice overuse. Symptoms of the common cold include urge to cough, hoarseness, pain on swallowing and dry mouth and throat. Patients in this study were requested to take one or two ectoine lozenges every 3 h or as needed.

Hyaluronic acid lozenges (GeloRevoice^®^ lozenges, G. Pohl-Boskamp GmbH & Co. KG, Hohenlockstedt, Germany) are also registered medical devices class I. Patients in this study were instructed to take one or two hyaluronic acid lozenges every 2–3 h and up to six times daily if necessary.

The hypertonic saline gargling solution was prepared by the patients at home by dissolving ¼ teaspoon of kitchen or table salt in a glass of warm water [29]. It was to be used three-to-five times daily.

### Clinical assessment

Investigators assessed three primary variables (pain on swallowing, urge to cough, hoarseness) and five secondary variables (dry mouth and throat, reddening of the oropharynx, reddening of the larynx, burning sensation in the throat, and patient’s general health condition) on a visual analogue scale from 0 (no symptoms) to 10 (severe symptoms) at V1 and V2. A sum score of three primary parameters ≥ 15 at V1 was defined as an inclusion criterion for patients’ participation in this study.

At the end of the study (V2), investigators assessed the general effectiveness and tolerability of the treatment as well as patient compliance on a visual analogue scale from 0 (very poor) to 10 (very good).

All above-mentioned variables, except for patient compliance and the symptoms of reddening of the oropharynx and the larynx, were also documented by the patients using the same scaling method in the patient diaries on a daily basis.

At V2, investigators also documented patients’ reports of any unpleasant oropharyngeal sensations perceived while applying the study treatment, patients’ concomitant use of oral ibuprofen and paracetamol, their likelihood of using the study treatment after the study, and their sick leaves.

All adverse events were to be documented by the investigators at V2.

### Statistical analyses

Data were analysed using the statistic software SAS^®^ version 9.3 (SAS Institute Inc., Cary, NC, USA). Demographic, medical history, and diagnostic data were evaluated descriptively. The frequency, mean, standard deviation, 95% confidence intervals, median, lower quartile, upper quartile, minimum, and maximum were calculated for each variable. Significant differences between V1 and V2 within a treatment group were determined using the Wilcoxon signed-rank test. Significant differences between treatment groups were detected using Chi square or Fisher’s exact test. Differences were considered statistically significant when *P* < 0.05. Finally, the Kaplan–Meier analysis was used to estimate the distribution of time to event.

## Results

### Study population

In all, 90 patients participated in this study as planned. Of those, 35 patients used the ectoine lozenges, 35 took the hyaluronic acid lozenges, and 20 applied the saline gargle. There were more female than male patients in all groups (ectoine group: 29 female, 6 male; hyaluronic acid group: 25 female, 10 male; saline gargle group: 13 female, 7 male); the distribution of female and male patients was similar across all groups. Patients taking ectoine lozenges had a mean age of 33.4 years, similar to that of 33.7 years in the hyaluronic acid group. The mean age of patients in the saline gargle group (49.4 years) was significantly higher than that of patients in the other two groups (*P* < 0.05). There were more smokers in the ectoine group than in the hyaluronic acid group and the saline gargle group (34.3%, 28.6%, and 25%, respectively) (Table 1).

**Table 1:** Demographic characteristics

Treatment	Ectoine lozenges	Hyaluronic acid lozenges	Saline gargle
Number of patients	35	35	20
Sex, *N* (%)			
Female	29 (82.9%)	25 (71.4%)	13 (65.0%)
Male	6 (17.1%)	10 (28.6%)	7 (35.0%)
Age, years			
Mean	33.4	33.7	49.4
SD	14.3	13.8	15.9
Minimum	18	18	21
Maximum	74	70	81
Smokers, *N* (%)	12 (34.3%)	10 (28.6%)	5 (25.0%)

*N* number, *SD* standard deviation

No adverse events or serious adverse events occurred during the study, and no patients dropped out. Complete data sets were obtained from all patients for all parameters.

### Improvement in pharyngitis symptoms

**Fig. 1:**
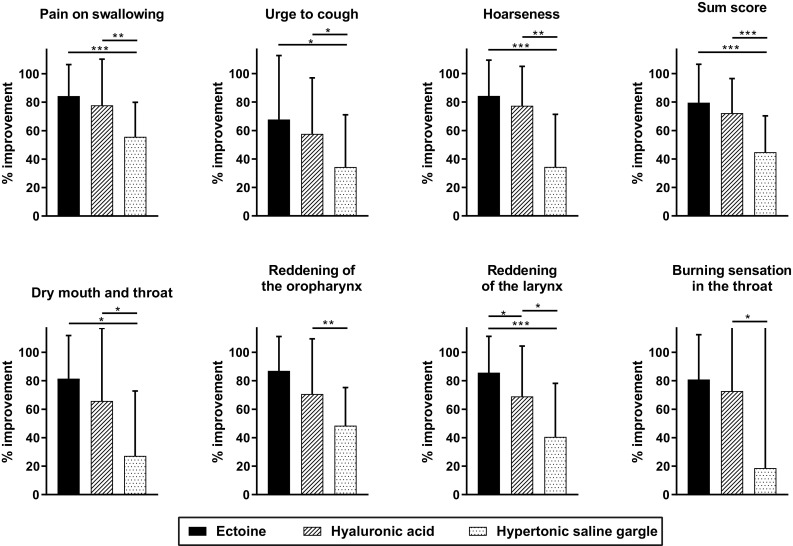
Reductions in pharyngitis symptom scores from V1 to V2 as assessed by the investigators. *P < 0.05, **P ≤ 0.001, ***P ≤ 0.0001

The greatest symptom improvement according to the investigators’ assessments was observed for the ectoine group, followed by that for the hyaluronic acid group and the saline gargle group. The sum score of the three primary variables (pain on swallowing, urge to cough, and hoarseness) in the ectoine group decreased significantly by 79.5% (V1: 19.93 ± 2.72; V2: 3.98 ± 5.26; *P* < 0.0001). In the hyaluronic acid group, the sum score decreased significantly by 72.2% (V1: 20.45 ± 3.01; V2: 5.77 ± 5.08; *P* < 0.0001) and in the saline gargle group by 44.8% (V1: 18.32 ± 2.42; V2: 9.89 ± 4.29; *P* < 0.0001). The reductions were significantly greater in the ectoine and the hyaluronic acid groups than in the saline gargle group (*P* < 0.05), whereas the difference between the ectoine and hyaluronic acid groups was not significant. With regard to individual symptoms, ectoine was superior to saline gargle in relieving all symptoms (*P* < 0.05). Hyaluronic acid was more advantageous than saline gargle in alleviating all symptoms (*P* < 0.05) except for reddening of the oropharynx and burning sensation in the throat. Ectoine was more effective than hyaluronic acid in ameliorating the symptom of reddening of the larynx (*P* < 0.05) (Fig. 1). Individual symptom scores and symptom improvement are summarised in the Online Supplemental Data Tables S1 and S2.

The symptom scores derived from patient diaries were mostly similar to the scores attained by the investigators. Throughout the study, the greatest symptom improvement was observed in patients treated with ectoine (Figure S1). Both ectoine and hyaluronic acids were significantly more effective than saline gargle in relieving most symptoms (*P* < 0.05); however, hyaluronic acid was not significantly better than saline gargle in relieving the symptom of pain on swallowing, and ectoine was not significantly better than saline gargle for the symptoms of urge to cough and burning sensation in the throat. The reductions in symptom scores were greater in the ectoine group than in the hyaluronic acid group, but the differences did not reach statistical significance.

The time efficiency was compared between the two lozenges using the Kaplan–Meier analysis of patients who attained a ≥ 50% recovery rate. Over the course of 7 days, the recovery rate was higher in the ectoine group than in the hyaluronic acid group. A strong effect was observed for the symptoms of pain on swallowing, hoarseness, and dry mouth and throat. However, our analyses did not yield a statistical significance between these two groups (Figure S3).

### Improvement in general health

**Fig. 2:**
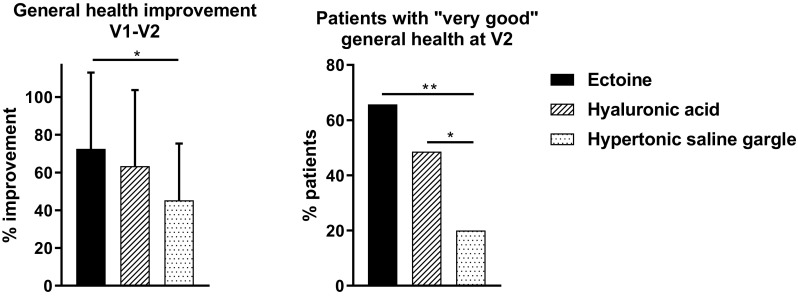
Improvement in patients’ general health as evaluated by the investigators. “Very good” general health = score < 2. *P < 0.05, **P ≤ 0.001

The investigators’ assessments resulted in a 72.5% improvement in the general health of patients in the ectoine group, thus greater than the improvement in the hyaluronic acid group (63.3%) and the saline gargle group (45.2%). Both the investigators’ and the patients’ assessments confirmed that ectoine was significantly more effective than saline gargle in improving patient’s general health condition (*P* < 0.05), but hyaluronic acid was not. Improvement in general health did not differ significantly between patients treated with ectoine and hyaluronic acid (Fig. 2; Table S1, Figure S1).

At the end of the study, 65.7% of patients treated with ectoine attained a “very good” general health condition (i.e., the score for general health < 2) in contrast to 48.6% of patients treated with hyaluronic acid and 20.0% with saline gargle according to the investigators. Statistical significance (*P* < 0.05) was shown when comparing ectoine with saline gargle and hyaluronic acid with saline gargle; patients treated with ectoine and hyaluronic acid did not differ significantly from each other (Fig. 2). The patients’ assessments yielded a relatively similar trend: 62.9% of patients in the ectoine group and 40.0% each in the hyaluronic acid group and the saline gargle group attained a “very good” general health condition on day 7. The difference between groups, however, was not significant (*P* = 0.094).

### General effectiveness, tolerability, and patient compliance

**Fig. 3:**
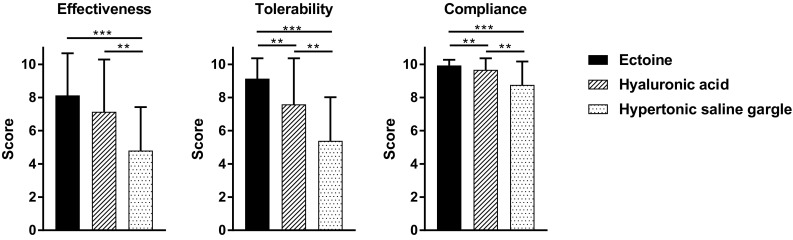
General effectiveness, tolerability, and patient compliance as assessed by the investigators. *P < 0.05, **P ≤ 0.001, ***P ≤ 0.0001

The investigators’ evaluation of the general effectiveness at V2 yielded a mean score of 8.13 ± 2.54 in the ectoine group, hence greater than that in the hyaluronic acid group (7.14 ± 3.15) and saline gargle group (4.80 ± 2.62). The difference between the ectoine and the hyaluronic acid group was not significant, but the differences between both of these groups and the saline gargle group were significant (*P* < 0.05) (Fig. 3). Data derived from patient diaries indicated the same results.

The score for the tolerability of ectoine lozenges as rated by the investigators reached a nearly perfect score (9.14 ± 1.22). The tolerability of ectoine was significantly better than that of hyaluronic acid (7.59 ± 2.77, *P* < 0.05). Both ectoine and hyaluronic acid were significantly more tolerable than was saline gargle (5.39 ± 2.63; *P* < 0.05) (Fig. 3). The analysis of the scores as documented by the patients yielded quite similar results: the mean tolerability score recorded on day 7 was 9.01 ± 1.35 in the ectoine group, 7.44 ± 3.14 in the hyaluronic acid group, and 5.39 ± 2.73 in the saline gargle group. The difference between the tolerability scores of ectoine and hyaluronic acid, however, was not significant (*P* = 0.064).

The compliance observed in patients treated with ectoine (9.93 ± 0.34) was significantly better than in those applying hyaluronic acid (9.67 ± 0.69; *P* < 0.05). Both lozenges were associated with significantly better patient compliance than the saline gargle (8.76 ± 1.41; *P* < 0.05) (Fig. 3).

### Other assessments

No patients using ectoine lozenges reported any unpleasant oropharyngeal sensations while applying them, whereas 40% of patients using hyaluronic acid lozenges and 45% of those who applied the saline gargle did. More patients in the ectoine group (23%) than in the hyaluronic acid (6%) and saline gargle (5%) groups said that they would continue their treatment after the study. Oral ibuprofen and paracetamol were used by 14% of patients in the ectoine group, 26% in the hyaluronic acid group, and 10% in the saline gargle group. Sick leave was prescribed for fewer patients in the ectoine group (17%) than in the hyaluronic acid group (40%); no patients using saline gargle took sick leave during the study.

## Discussion

Topical formulations for the treatment of pharyngitis include gargles, sprays, and lozenges. Among those, lozenges provide more targeted and longer availability of active ingredients in the pharyngeal cavity [30]. Compared to systemic administration, the advantage of topical treatment is the direct delivery of the active substance to the inflamed area, thus reducing the risk of toxicity. Topical treatment of pharyngitis includes the use of non-steroidal anti-inflammatory drugs (e.g., flurbiprofen and ibuprofen) [31, 32], oral/intramuscular steroids (e.g., dexamethasone, betamethasone, and prednisone) [33, 34], AMC/DCBA (e.g., Strepsils^®^) [35], and ambroxol [36, 37]. Although all of the above clinical trials yielded positive results, it is noteworthy that topical anaesthetics or antiseptics are not recommended in the guidelines for the management of sore throat by the German Society of General Practice and Family Medicine, since such treatments usually cannot shorten the duration of inflammation [8]. According to the guidelines, natural therapeutic agents, home remedies, and over-the-counter products should be used with care, since they may cause untoward drug interactions and few clinical trials are available demonstrating their efficacy.

In this study, patients were treated with either ectoine lozenges, hyaluronic acid lozenges, or hypertonic saline gargle. In general, gargling is a common home remedy for cleansing the throat, and gargling with hypertonic saline solution has long been used, because it is known for its sore throat alleviation properties [38]. In 2015, a randomised clinical trial demonstrated that hypertonic saline irrigation and gargling for the common cold significantly reduce symptom duration and severity as well as over-the-counter medication use [39]. Furthermore, several Cochrane reviews summarise the evidence for the positive effects of hypertonic saline solution in the management of the symptoms of upper respiratory tract infections [40–42].

Ectoine lozenges and hyaluronic acid lozenges are over-the-counter products containing a natural therapeutic ingredient. In this study, analyses of the symptom scores, patients’ general health, and general treatment effectiveness implied numerical superiority of ectoine over hyaluronic acid. In a previous study which showed hyaluronic acid lozenges to be superior to saline gargle and lozenges containing Icelandic moss, hyaluronic acid lozenges improved pain on swallowing by 77.4%, urge to cough by 75.0%, and hoarseness by 77.4% after 7 days of treatment [43]. This is in line with the symptom improvement observed in the hyaluronic acid group of the present study.

Notably, tolerability and patient compliance were significantly better for ectoine lozenges than for hyaluronic acid lozenges. The excellent tolerability and safety profile of ectoine [44] is one of its advantages in topical use. While almost half of the patients receiving hyaluronic acid lozenges reported unpleasant experiences, no patients treated with ectoine lozenges did. The slightly effervescent effect of the hyaluronic acid lozenges is key for the thorough distribution of medication within the mouth and throat area; it is likely that patients found this effervescent, salivation-increasing experience to be unpleasant. In contrast, ectoine lozenges work by slowly dissolving in the pharyngeal cavity. Sucking on lozenges also stimulates saliva production, thereby lubricating and soothing irritated throat linings. Our results demonstrated that ectoine lozenges are more effective than hyaluronic acid lozenges in improving dry mouth and throat. Therefore, it seems ectoine lozenges also effectively increase saliva production without involving any unpleasant effervescent effects.

Results of this study permit the conclusion that ectoine lozenges are superior to hyaluronic acid lozenges and hypertonic saline gargle in the treatment of acute viral pharyngitis. Of note, hyaluronic acid lozenges have been the best-selling product for sore throat on the German market since 2009 [45]. The comparable demographic and baseline characteristics between the two lozenge groups provided validity for these outcomes. Unlike the two lozenge groups, the saline gargle group consisted of older patients with slightly milder symptoms. Thus, the comparison between the two lozenge groups could be considered the primary outcome of this study. Nevertheless, analyses of all tested variables consistently demonstrated that both lozenges were superior to hypertonic saline gargle.

The strengths of this study were its prospective, active-controlled, patient-preference design, which reflects real-life medical practice, and its use of well-established instruments such as the visual analogue scale. The drawback of this study design was the lack of randomisation, resulting in more older patients having less severe symptoms who chose the home remedy saline gargle. The lack of a placebo control was also a weakness of this study, which is an inherent problem in pharyngitis studies involving topical treatment with lozenges.

## Conclusions

This study, performed in a real-life setting, showed that treatment with ectoine lozenges is more effective and tolerable than treatments with hyaluronic lozenges and hypertonic saline gargle in patients with moderate-to-severe symptoms of acute viral pharyngitis.

## Electronic supplementary material

Below is the link to the electronic supplementary material.

Table S1: Symptom scores. CI, confidence interval; SD, standard deviation; V, visit (DOCX 17 KB)

Table S2: Symptom improvements (%). CI, confidence interval; SD, standard deviation; V, visit (DOCX 15 KB)

Figure S1: Patient-reported symptom improvements (%) (baseline = day 1) (EPS 65 KB)

Figure S2: Kaplan–Meier analysis of patients who attained ≥ 50% improvement (baseline = day 1) (EPS 18 KB)

## Abbreviations

MPG: Medizinproduktegesetz (German Medical Devices Act)
*N*: Number
SD: Standard deviation
V: Visit
