# Supplementary material for: Ectoine lozenges in the treatment of acute viral pharyngitis: a prospective, active-controlled clinical study
Source: Eur Arch Otorhinolaryngol. 2019 Feb 9;276(3):775–83. doi: 10.1007/s00405-019-05324-9 (PMC6411829; doi:10.1007/s00405-019-05324-9)
Supplement: Supplementary file 1 — Table S1: Symptom scores. CI, confidence interval; SD, standard deviation; V, visit (DOCX 17 KB) [file 405_2019_5324_MOESM1_ESM.docx]

**Table S1**

| Parameter | V | Treatment | Mean | 95% CI | | SD | Median |
| --- | --- | --- | --- | --- | --- | --- | --- |
| Pain on swallowing | V1 | Ectoine | 7.43 | 7.02 | 7.84 | 1.24 | 7.70 |
|  |  | Hyaluronic acid | 7.39 | 6.95 | 7.83 | 1.34 | 7.90 |
|  |  | Saline gargle | 7.04 | 6.62 | 7.46 | 0.95 | 7.45 |
|  | V2 | Ectoine | 1.18 | 0.61 | 1.75 | 1.72 | 0.20 |
|  |  | Hyaluronic acid | 1.62 | 0.88 | 2.37 | 2.24 | 1.10 |
|  |  | Saline gargle | 3.15 | 2.38 | 3.91 | 1.76 | 3.15 |
| Urge to cough | V1 | Ectoine | 6.23 | 5.75 | 6.71 | 1.46 | 6.50 |
|  |  | Hyaluronic acid | 6.97 | 6.42 | 7.52 | 1.66 | 7.40 |
|  |  | Saline gargle | 5.76 | 5.18 | 6.34 | 1.31 | 5.60 |
|  | V2 | Ectoine | 1.88 | 1.00 | 2.76 | 2.65 | 0.60 |
|  |  | Hyaluronic acid | 2.73 | 2.00 | 3.46 | 2.20 | 2.40 |
|  |  | Saline gargle | 3.53 | 2.77 | 4.29 | 1.73 | 3.25 |
| Hoarseness | V1 | Ectoine | 6.27 | 5.56 | 6.98 | 2.14 | 6.10 |
|  |  | Hyaluronic acid | 6.09 | 5.35 | 6.83 | 2.23 | 5.80 |
|  |  | Saline gargle | 5.52 | 4.63 | 6.41 | 2.02 | 5.30 |
|  | V2 | Ectoine | 0.96 | 0.40 | 1.52 | 1.70 | 0.50 |
|  |  | Hyaluronic acid | 1.42 | 0.88 | 1.95 | 1.61 | 0.80 |
|  |  | Saline gargle | 3.22 | 2.50 | 3.94 | 1.65 | 2.95 |
| Sum score | V1 | Ectoine | 19.93 | 19.03 | 20.83 | 2.72 | 20.00 |
|  |  | Hyaluronic acid | 20.45 | 19.46 | 21.45 | 3.01 | 19.90 |
|  |  | Saline gargle | 18.32 | 17.26 | 19.38 | 2.42 | 17.50 |
|  | V2 | Ectoine | 3.98 | 2.24 | 5.73 | 5.26 | 2.00 |
|  |  | Hyaluronic acid | 5.77 | 4.09 | 7.45 | 5.08 | 4.00 |
|  |  | Saline gargle | 9.89 | 8.02 | 11.77 | 4.29 | 9.20 |
| Dry mouth and throat | V1 | Ectoine | 5.82 | 4.75 | 6.88 | 3.21 | 6.50 |
|  |  | Hyaluronic acid | 5.68 | 4.89 | 6.47 | 2.39 | 6.50 |
|  |  | Saline gargle | 4.28 | 2.90 | 5.65 | 3.13 | 2.70 |
|  | V2 | Ectoine | 1.41 | 0.52 | 2.30 | 2.68 | 0.00 |
|  |  | Hyaluronic acid | 1.69 | 0.99 | 2.40 | 2.13 | 0.90 |
|  |  | Saline gargle | 2.52 | 1.57 | 3.46 | 2.15 | 2.10 |
| Reddening of the oropharynx | V1 | Ectoine | 5.92 | 5.32 | 6.51 | 1.80 | 5.50 |
|  |  | Hyaluronic acid | 5.50 | 4.89 | 6.11 | 1.84 | 5.10 |
|  |  | Saline gargle | 5.49 | 4.73 | 6.24 | 1.72 | 5.05 |
|  | V2 | Ectoine | 0.80 | 0.35 | 1.26 | 1.37 | 0.00 |
|  |  | Hyaluronic acid | 1.43 | 0.89 | 1.98 | 1.65 | 0.70 |
|  |  | Saline gargle | 2.67 | 1.97 | 3.36 | 1.60 | 2.60 |
| Reddening of the larynx | V1 | Ectoine | 6.27 | 5.73 | 6.82 | 1.65 | 6.00 |
|  |  | Hyaluronic acid | 5.79 | 5.14 | 6.45 | 1.98 | 5.60 |
|  |  | Saline gargle | 5.53 | 4.78 | 6.28 | 1.71 | 5.70 |
|  | V2 | Ectoine | 0.79 | 0.35 | 1.22 | 1.32 | 0.00 |
|  |  | Hyaluronic acid | 1.56 | 1.03 | 2.09 | 1.60 | 1.50 |
|  |  | Saline gargle | 2.87 | 2.30 | 3.44 | 1.29 | 2.80 |
| Burning sensation in the throat | V1 | Ectoine | 5.37 | 4.51 | 6.22 | 2.58 | 5.60 |
|  |  | Hyaluronic acid | 4.56 | 3.67 | 5.46 | 2.71 | 4.60 |
|  |  | Saline gargle | 4.50 | 3.28 | 5.72 | 2.79 | 3.45 |
|  | V2 | Ectoine | 0.87 | 0.31 | 1.43 | 1.69 | 0.00 |
|  |  | Hyaluronic acid | 1.15 | 0.43 | 1.86 | 2.15 | 0.00 |
|  |  | Saline gargle | 2.16 | 1.39 | 2.92 | 1.75 | 2.25 |
| Patient's general health | V1 | Ectoine | 6.34 | 5.64 | 7.05 | 2.12 | 6.00 |
|  |  | Hyaluronic acid | 6.58 | 5.89 | 7.27 | 2.07 | 6.80 |
|  |  | Saline gargle | 5.64 | 4.94 | 6.34 | 1.59 | 5.20 |
|  | V2 | Ectoine | 1.71 | 0.98 | 2.45 | 2.22 | 0.70 |
|  |  | Hyaluronic acid | 2.33 | 1.59 | 3.07 | 2.24 | 2.00 |
|  |  | Saline gargle | 2.81 | 2.11 | 3.51 | 1.59 | 2.90 |
