# Supplementary material for: Ectoine lozenges in the treatment of acute viral pharyngitis: a prospective, active-controlled clinical study
Source: Eur Arch Otorhinolaryngol. 2019 Feb 9;276(3):775–83. doi: 10.1007/s00405-019-05324-9 (PMC6411829; doi:10.1007/s00405-019-05324-9)
Supplement: Supplementary file 2 — Table S2: Symptom improvements (%). CI, confidence interval; SD, standard deviation; V, visit (DOCX 15 KB) [file 405_2019_5324_MOESM2_ESM.docx]

**Table S2**

| Parameter | Treatment | Mean | 95% CI | | SD | Median | *P* |
| --- | --- | --- | --- | --- | --- | --- | --- |
| Pain on swallowing | Ectoine | 84.35 | 77.04 | 91.66 | 22.06 | 97.56 | <0.0001 |
|  | Hyaluronic acid | 77.78 | 67.04 | 88.52 | 32.42 | 86.21 | <0.0001 |
|  | Saline gargle | 55.72 | 45.10 | 66.34 | 24.23 | 55.00 | <0.0001 |
| Urge to cough | Ectoine | 67.75 | 52.88 | 82.61 | 44.87 | 84.75 | <0.0001 |
|  | Hyaluronic acid | 57.59 | 44.53 | 70.65 | 39.41 | 67.92 | <0.0001 |
|  | Saline gargle | 34.37 | 18.27 | 50.47 | 36.74 | 41.55 | 0.0007 |
| Hoarseness | Ectoine | 84.30 | 75.98 | 92.62 | 25.11 | 93.75 | <0.0001 |
|  | Hyaluronic acid | 77.42 | 68.28 | 86.56 | 27.60 | 86.21 | <0.0001 |
|  | Saline gargle | 34.40 | 18.20 | 50.60 | 36.97 | 45.32 | 0.0005 |
| Sum score | Ectoine | 79.54 | 70.60 | 88.47 | 26.98 | 89.47 | <0.0001 |
|  | Hyaluronic acid | 72.21 | 64.17 | 80.25 | 24.26 | 79.17 | <0.0001 |
|  | Saline gargle | 44.78 | 33.61 | 55.95 | 25.48 | 46.99 | <0.0001 |
| Dry mouth and throat | Ectoine | 81.45 | 71.09 | 91.82 | 30.37 | 100.00 | <0.0001 |
|  | Hyaluronic acid | 65.84 | 48.44 | 83.24 | 51.00 | 83.02 | <0.0001 |
|  | Saline gargle | 27.18 | 6.65 | 47.70 | 45.65 | 41.18 | 0.0144 |
| Reddening of the oropharynx | Ectoine | 86.93 | 78.90 | 94.95 | 24.22 | 100.00 | <0.0001 |
|  | Hyaluronic acid | 70.65 | 57.80 | 83.51 | 38.79 | 78.26 | <0.0001 |
|  | Saline gargle | 48.43 | 36.67 | 60.19 | 26.84 | 47.06 | <0.0001 |
| Reddening of the larynx | Ectoine | 85.68 | 77.24 | 94.12 | 25.48 | 100.00 | <0.0001 |
|  | Hyaluronic acid | 69.02 | 57.30 | 80.73 | 35.37 | 78.41 | <0.0001 |
|  | Saline gargle | 40.62 | 24.15 | 57.09 | 37.57 | 53.43 | 0.0006 |
| Burning sensation in the throat | Ectoine | 80.86 | 69.94 | 91.79 | 31.54 | 100.00 | <0.0001 |
|  | Hyaluronic acid | 72.76 | 53.24 | 92.28 | 56.34 | 99.50 | <0.0001 |
|  | Saline gargle | 18.70 | -28.32 | 65.71 | 107.27 | 50.45 | 0.0401 |
| Patient's general health | Ectoine | 72.53 | 59.12 | 85.93 | 40.47 | 91.11 | <0.0001 |
|  | Hyaluronic acid | 63.33 | 49.94 | 76.72 | 40.42 | 70.69 | <0.0001 |
|  | Saline gargle | 45.23 | 32.04 | 58.41 | 30.09 | 37.63 | <0.0001 |
